# A Genetic Link to Obesity: The Numbers Don't Add Up for *GAD2*

**DOI:** 10.1371/journal.pbio.0030321

**Published:** 2005-08-30

Obesity is a leading cause of preventable death and is often linked to type II diabetes and heart disease. Being a complex trait, obesity is likely caused by the interplay of multiple environmental factors and many genes. Common genetic differences between individuals within a region of Chromosome 10 have previously been associated with obesity. This region contains several genes with the potential to be directly involved in the disease. One of these genes, *GAD2*, has been the subject of many studies. A new study by Michael Swarbrick, Björn Waldenmaier, Christian Vaisse, and their colleagues takes a new look at *GAD2* and provides strong evidence that the gene might not be as relevant to obesity as previously thought.

*GAD2* encodes a protein (called GAD-65) involved in the production of GABA, a neurotransmitter involved in a variety of brain functions, including appetite stimulation and energy consumption. Studies in mice have shown that increased levels of GABA result in hunger and overeating. In healthy mice, the levels of *GAD2*, and hence, GABA, are controlled, making sure that the balance between weight gain and loss is maintained. A 2003 study of a French population found that three genetic mutations in and around the *GAD2* gene occurred at a high level in individuals with obesity. The 2003 study, conducted by different researchers, was also published in *PLoS Biology*. When Swarbrick et al. surveyed German, Caucasian-American, and Canadian populations for this genetic correlation, however, they found no statistically significant link between obesity and any of the mutations. [Fig pbio-0030321-g001]

**Figure pbio-0030321-g001:**
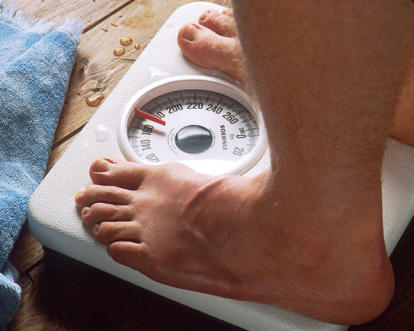
Scientists believe that genetic mutations in a specific region in Chromosome 10 play a role in obesity and have studied one gene, *GAD2*, intensively. But a new study finds no evidence linking *GAD2* mutations with obesity

There are many possible reasons why different studies may show different results: ethnic differences between populations, as well as behavioral and dietary differences, could account for varying results when it comes to studying a trait as complex as obesity. Also, studies that seek to show an association between genetic differences and complex diseases rely heavily on the statistical power of their tests, which depends on the number of subjects involved. Swarbrick et al. have not only studied 2,359 German, 729 US, and 1,137 Canadian subjects, but also conducted a “meta-analysis”—a statistical analysis of a collection of individual studies—of their data and the previously published data from 1,221 French subjects. Meta-analyses help identify patterns from multiple individual studies that may not be visible in any one study alone, and also help rule out chance differences that may be apparent in one single study. In this case, the meta-analysis showed that when the results from French subjects are put together with the results from other ethnic populations, there is no evidence for a link between changes in *GAD2* and obesity.

Although *GAD2*'s role in controlling appetite made it an exciting candidate for a link to obesity-related conditions, Swarbrick et al. show that the numbers simply don't add up. The search for serious obesity gene contenders in this region of Chromosome 10 is all set to continue—and attention can now turn to several other potential gene candidates located nearby.

